# AI-assisted deep learning segmentation and quantitative analysis of X-ray microtomography data from biomass ashes

**DOI:** 10.1016/j.mex.2024.102812

**Published:** 2024-06-24

**Authors:** Anna Strandberg, Hubert Chevreau, Nils Skoglund

**Affiliations:** a Umeå University, Department of Applied Physics and Electronics, Thermochemical Energy Conversion Laboratory, SE-901 87 Umeå, Sweden; b Synchrotron SOLEIL, L Orme des Merisiers, Saint-Aubin, BP 48, 91192 Gif-sur-Yvette, France

**Keywords:** Micro-CT, µCT, Image analysis, Internal microstructure, Porosity, Open pore volume, Pore-size distribution, Wall thickness, Specific surface area, ash recycling, Image analysis of X-ray tomography data with deep learning segmentation and quantitative analysis

## Abstract

X-ray microtomography is a non-destructive method that allows for detailed three-dimensional visualisation of the internal microstructure of materials. In the context of using phosphorus-rich residual streams in combustion for further ash recycling, physical properties of ash particles can play a crucial role in ensuring effective nutrient return and sustainable practices. In previous work, parameters such as surface area, porosity, and pore size distribution, were determined for ash particles. However, the image analysis involved binary segmentation followed by time-consuming manual corrections. The current work presents a method to implement deep learning segmentation and an approach for quantitative analysis of morphology, porosity, and internal microstructure. Deep learning segmentation was applied to microtomography data. The model, with U-Net architecture, was trained using manual input and algorithm prediction.

• The trained and validated deep learning model could accurately segment material (ash) and air (pores and background) for these heterogeneous particles.
• Quantitative analysis was performed for the segmented data on porosity, open pore volume, pore size distribution, sphericity, particle wall thickness and specific surface area.
• Material features with similar intensities but different patterns, intensity variations in the background and artefacts could not be separated by manual segmentation – this challenge was resolved using the deep learning approach.

## Graphical abstract

**Figure:**
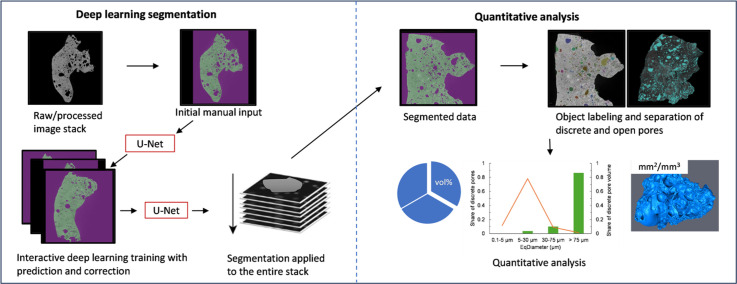

Specifications table

**Table:** 

Subject area:	Materials Science
More specific subject area:	Image analysis, deep learning segmentation and quantitative data analysis
Name of your method:	Image analysis of X-ray tomography data with deep learning segmentation and quantitative analysis
Name and reference of original method:	X-ray microtomography and image analysis: A. Strandberg, N. Skoglund, M. Thyrel, Morphological characterisation of ash particles from co-combustion of sewage sludge and wheat straw with X-ray microtomography, Waste Management 135 (2021) 30–39. https://www.sciencedirect.com/science/article/pii/S0956053X21004517
Resource availability:	Dragonfly software, Version 2022.2 for Windows, Object Research Systems (ORS) Inc, Montreal, Canada; (software available at http://www.theobjects.com/dragonfly)

## Background

X-ray microtomography is a technique for 3D imaging and non-destructive characterisation of materials. With 3D image analysis, quantitative characterisation of morphology and internal microstructure can be performed based on the material's X-ray attenuation, which varies depending on density and atomic composition [[1], [2], [3]]. AI training using deep learning on tomography data can enhance the data analysis; others have successfully done this [4,5].

In the context of using phosphorus-rich residual streams in combustion for further ash recycling, the physical properties of ash particles can play a crucial role in ensuring effective nutrient return and sustainable practices. While research on the influence of ash particles on soil is limited, insights from studies on biochars can provide valuable comparisons. The porosity of ash particles, including pore volume and size distribution, is particularly significant regarding leaching and water-holding capacity [6,7]. The pore size distribution in soil affects its ability to transmit and store water. Understanding these pore characteristics in ash particles and their impact on soil can contribute to optimising nutrient release and water management in agricultural practices.

In previous work (Strandberg et al. [8], Skoglund et al. [9]), the specific surface area, porosity, and pore size distribution for different ash particles were determined. However, the image analysis was built on manual binary segmentation, a method that proved to be time-consuming. Within this context, the aim of the present work was to implement deep learning segmentation and an approach for quantitative analysis of morphology, porosity, and internal microstructure of the samples, thereby enhancing the efficiency and accuracy of our research.

## Method details

### Material

The material in this project is sintered ash particles (slag) from bottom ash after fixed bed-combustion of phosphorus-rich fuel mixtures. After combustion, these nutrient-containing bottom ash fractions often contain both fine powdery material and sintered ash particles (slag). The sintered ash comprises particles that have been molten, at least partially, leading to forming internal pores and encapsulating crystal compounds in a non-crystalline matrix. Description of fuel properties, combustion experiments and ash characterisation has previously been described by Skoglund et al. [9] and Falk et al. [10].

### X-ray microtomography and data analysis

Microtomographic scans were performed at the beamline ANATOMIX at the Synchrotron SOLEIL, France [11]. The polychromatic ('white') X-ray beam had a central photon energy of around 40 keV with an electron beam current of 100 mA. A digital camera (ORCA Flash 4.0 V2), with a 2X magnification lens and an exposure time of 10–50 ms was used for each image. The field of view was 6.5 mm, and the pixel resolution was 3.07 µm. 1600–5900 projections were collected while the sample rotated over a 180–360° range, including extended-field tomography for samples not fitted in the field of view.

The reconstruction software PyHST2 [12], which uses the filtered back-projection algorithm, was used for reconstruction. For decreasing ring artefacts, a double flatfield algorithm was applied, and a Paganini filter was used for semi-quantitative phase retrieval [13,14].

The data analysis, images and 3D visualisations were generated using Dragonfly software under a free academic license (Version 2022.2 for Windows, Object Research Systems (ORS) Inc, Montreal, Canada; software available at http://www.theobjects.com/dragonfly). The segmentation wizard within Dragonfly was used to label the tomographic images to create a database as well as the training and validation of the deep learning model. All models were created with native toolkits. The computer was equipped with a dedicated Nvidia RTX A5000 Graphics Processing Unit with 24 GB and a RAM of 384 GB.

### Data analysis methods

#### Deep learning segmentation

Deep learning segmentation was applied to X-ray microtomography data. The model was trained using manual input and algorithm prediction, and its accuracy was evaluated using a validation loss function.

**Fig. 1:**
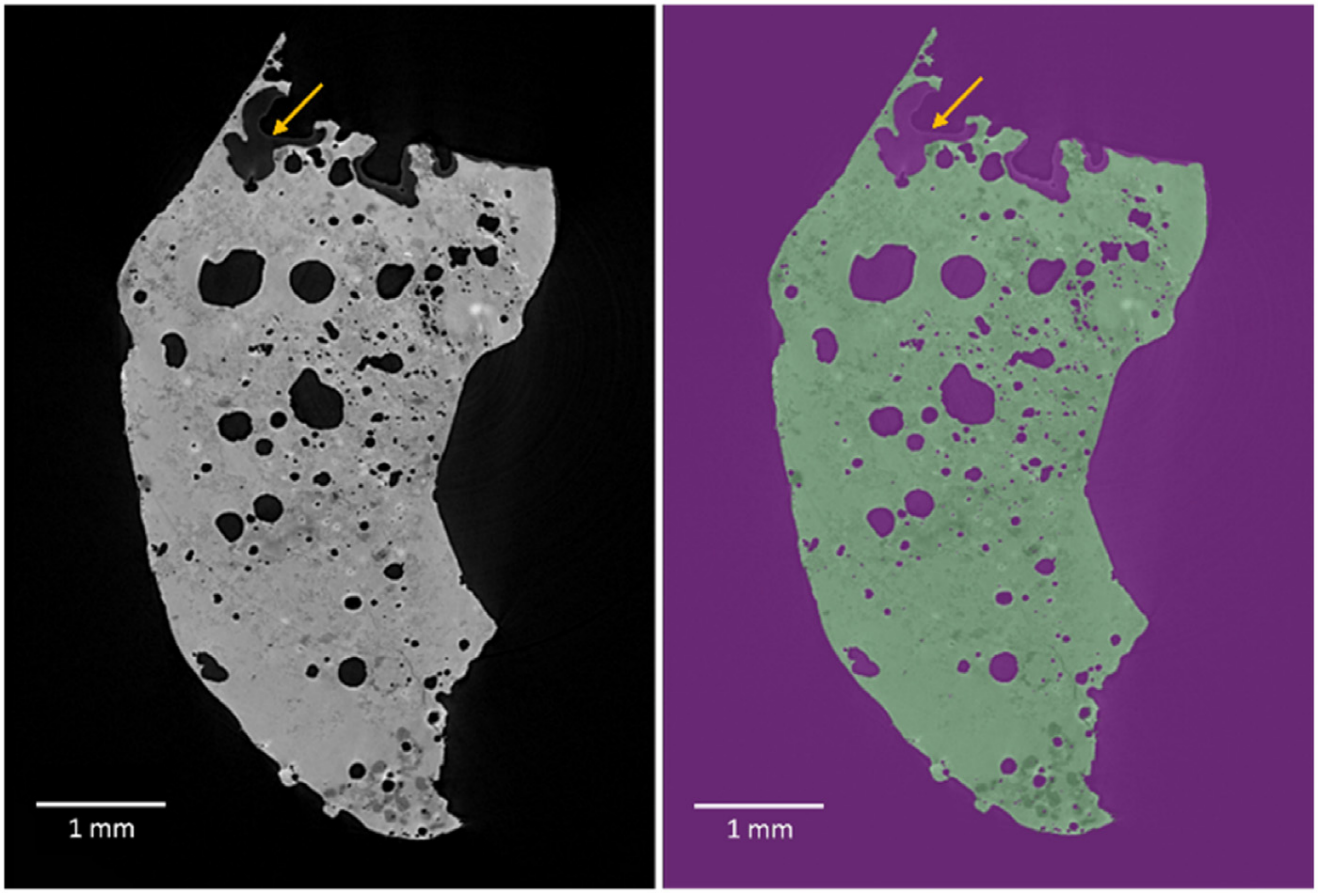
An ortho slice, showing the cross-section of the sample, with the grey scale apply to X-ray attenuation intensities to the left and the labelled one used for model training to the right, where green is the ash and purple the air. In the top of the ash particle, some glue can be seen, indicated with an arrow.

Segmentation of the data is necessary to enable quantitative analyses. In this particular study, the images were segmented as solid material (ash) and air (pores and background), as shown in Fig. 1. These samples are heterogeneous and complex, with a large open pore volume that needs to be accurately distinguished from the background. Thresholding based solely on attenuation doesn't work very well, and manual adjustments are time-consuming. Additionally, the presence of glue from the mounting process during data collection, which shares a similar intensity with some parts of the sample but has a clear difference in pattern, further complicated the segmentation process; the glue can be seen at the top of Fig. 1. There were also slight intensity variations between the centre and the top, respective bottom of the sample, as well as intensity variation in the background and minor ring artefacts. These inherent sample complexities necessitate implementation of deep learning segmentation. While, in this case, the data was only segmented into ash and air, the same method could potentially be used to segment more features.

A dataset was first created by manual labelling using the Segmentation Wizard, then a deep learning model with a U-net architecture based on a convolutional network was trained and validated. A depth level of 4 layers was used with an initial filter count of 64 [15]. The input dimension was 2.5D, and the input slice count was 3. The training data for the segmentation model was formed by painting (labelling) some frames (ortho slices) in the X-ray microtomography data by manual input. More training data was generated by empowering the deep learning algorithm to predict results in additional frames, which were then manually corrected. The final labelled dataset containing 10 frames was used to train a U-net model, the inference was then performed on the entire volume stack. The training parameters were 100 epochs, a stride ratio 0.25, and a batch size 512, using the optimisation algorithm ADADELTA [16]. The validation loss function ORS categorical cross-entropy was used to evaluate the model by assessing its accuracy by comparing the training dataset to the validation dataset. 20 % of the dataset was used for validation. The training was aborted if there was no improvement in validation loss for 15 consecutive epochs.

**Fig. 2:**
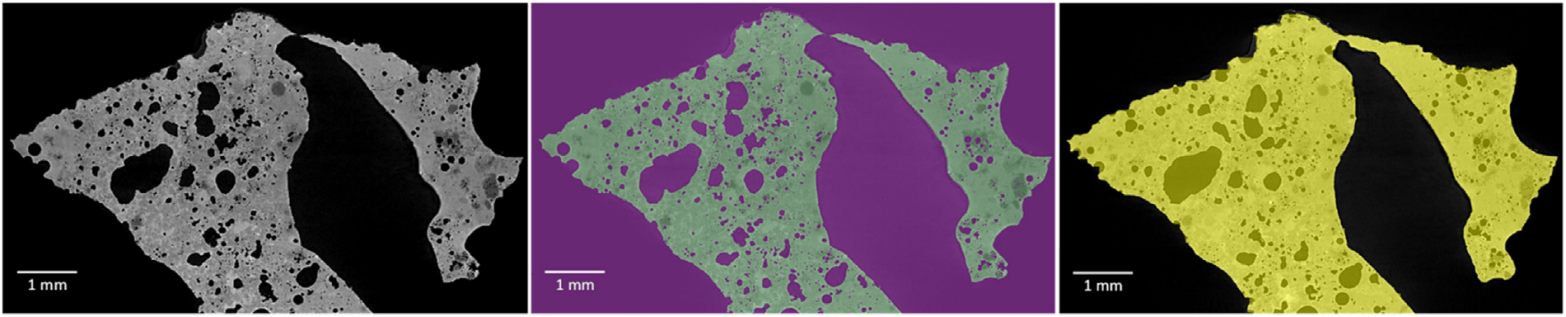
An ortho slice with grey scale according to X-ray attenuation intensities to the left, the segmented model in the middle with the ROI “Ash Particle” as green and ROI “Air” as violet, and to the right a ROI “Filled particle” in yellow.

The trained model was then applied to the whole dataset by using the function segment with AI. In this case, reference slices 2 and spacing 1 were used. A region of interest (ROI) for the ash, named “Ash particle”, and air, named “Air”, was created (Fig. 2). To avoid small dust/trash coming along with the ash, only the large particle is saved by using a connected component and extracting the largest class as ROI. In the case of several particles, the smallest can be excluded in an equivalent way.

## Object labelling and separation of pores

### Separation of pores and background

After segmentation, the ROI “Air” was separated into background and pores by the following steps:

(1) A copy of the ROI “Ash particle” was chosen as a new ROI named “Filled particle”. The morphological operation “Close” was applied on orthogonal slices in dimension 2D (X), 2D (Y), and 2D (Z) directions, with a kernel size 33, and the operation “Fill inner areas” was used for 2D (X), 2D (Y), and 2D (Z) direction. An ortho slice from the resulted ROI can be seen to the right in Fig. 2.
  Important quality check: Review the ROI so it appears representative for expected features. The kernel size may need to be adjusted. The connected component function can be used, and any loose parts can be removed.
(2) The region not belonging to the “Filled particle” was assigned to a new ROI “Background”.
(3) The ROI “Background” and the ROI “Ash particle” was merged into a multi-ROI. In this multi-ROI, another ROI was created, named “Pores”. All voxels not assigned to a class in this multi-ROI were added to the “Pores”, which now consists of all pores.

As a result of these steps, there is now one ROI “Ash Particle”, one “Pores”, and one “Background”. Using the connected component function on the ROI “Pores” generated a new multi-ROI, with each pore space having a separate identity.

### Separation of discrete and open pores

**Fig. 3:**
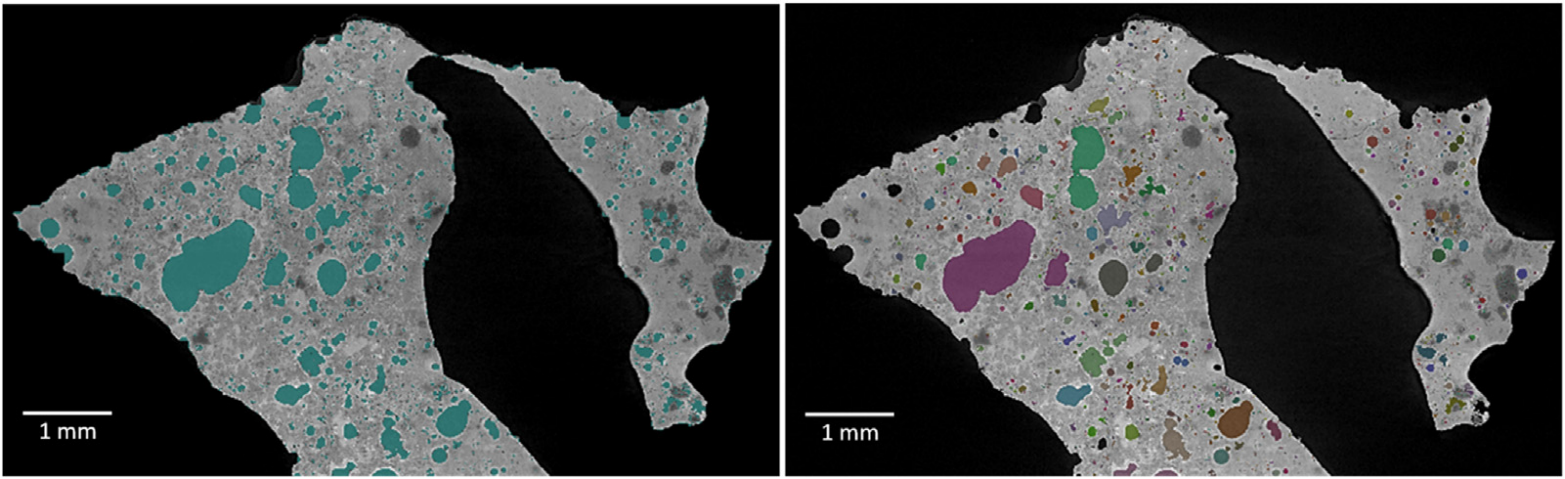
An ortho slice showing, all pores to the left and, to the right, the discrete pores, (coloured after pore size).

Pores were classified as either *Open* – i.e. have physical linkage to the surrounding volume, or *Discrete* – i.e. be a self-contained volume enclosed by the ROI Ash Particle. By using the function connected component on the ROI “Air”, a new multi-ROI was generated that includes all pores and background together, but as separate components. By removing the largest component (surrounding background and the connected pores), the discrete pores are left. The ROI was named “Discrete pores”. Fig. 3 shows all pores (left) and only the discrete pores (right).

## Quantitative analysis

### Porosity and open pore volume

Porosity refers to the presence of voids or empty spaces within a material. The volume measurement of the ROI “Ash particle” respective “Pores” was used. The porosity was calculated by dividing the volume of the “Pores” by the sum of the total volume consisting of “Pores” and “Ash particle”.

The volume of the discrete pores was then obtained from the ROI “Discrete pores”. The open pore volume was calculated by subtracting the “Discrete pores” volume from the “Pores”.

### Pore size distribution and sphericity

The scalar generator was used to statistically analyse the pores with basic measurements for volume, equivalent spherical diameter, surface area (Lindblad 2005, [17]), and sphericity. The statistical dataset was then exported to CSV files for further analysis, using, for example, Matlab or Excel. Volume colouring can be used for visualisation.

The pore size distribution was generated using the equivalent spherical diameter, *D_V_. D_V_* is defined as the diameter of an equivalent sphere whose volume equals that of the pore, given by the following formula [18]:

(1) $$D_{V}\;=\;\sqrt[{3}]{6\;\times V_{P}/\pi },$$

where D_V_ is the diameter of a volume-equivalent sphere, and V_P_ is the volume of the pore.

**Fig. 4:**
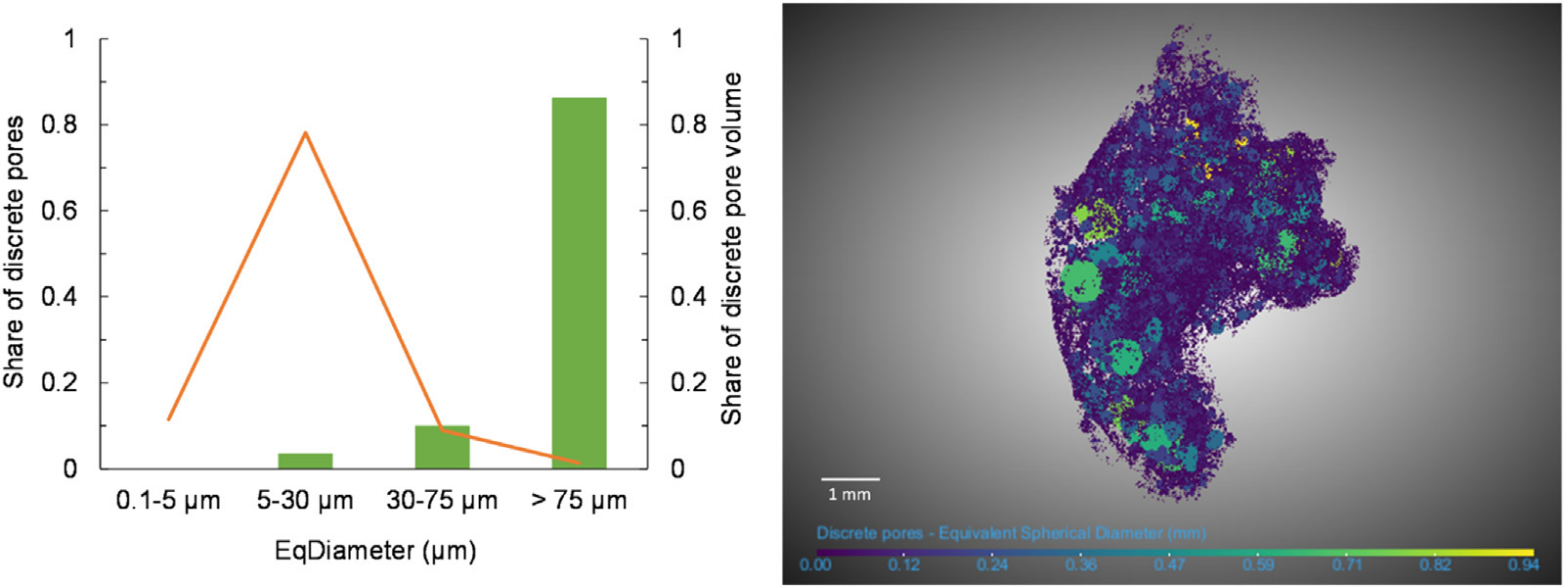
Pore size distribution for the discrete pores. To the left is a graph with the share of numbers of pores marked with an orange line and the share of pore volume marked with columns. To the right is a 3D representation of the distribution and size (equivalent diameter) of discrete pores, coloured after pore size.

Histograms with size classes can represent the pore size distribution. The pore size distribution in Fig. 4 (left) is presented as the share of the number of discrete pores (Y1) and the share of the discrete pore volume (Y2). This was calculated as the sum of discrete pores per size range divided by the total number of discrete pores, respective as the sum of the discrete pore volume per size range divided by the total discrete pore volume. It is worth noticing that the pores here were divided into classes, according to the definition of the Soil Science Society of America [19] and used by Cameron and Buchan [20]. The smallest pores in the dataset have an equivalent diameter of 3.8 µm as a limitation of the resolution. With a higher resolution, the smallest class would probably have more pores.

Fig. 4 (right) demonstrates another approach to represent the size and 3D distribution of pores, with a 3D reconstruction of the discrete pores, which was achieved through colouring the pores in the multi-ROI “Discrete pores” after equivalent diameter.

The sphericity can be estimated by taking the surface area of an equivalent sphere with the same volume as the particle studied and dividing that with the surface area of the studied particle [18], and calculated by:

(2) $$\varphi \;=\pi^{1/3}{\left(6\;\times V_{P}\right)}^{2/3}/A_{P}$$

where ϕ is the sphericity, V_P_ is the volume, and A_P_ is the surface area of the pore.

### Particle wall thickness

**Fig. 5:**
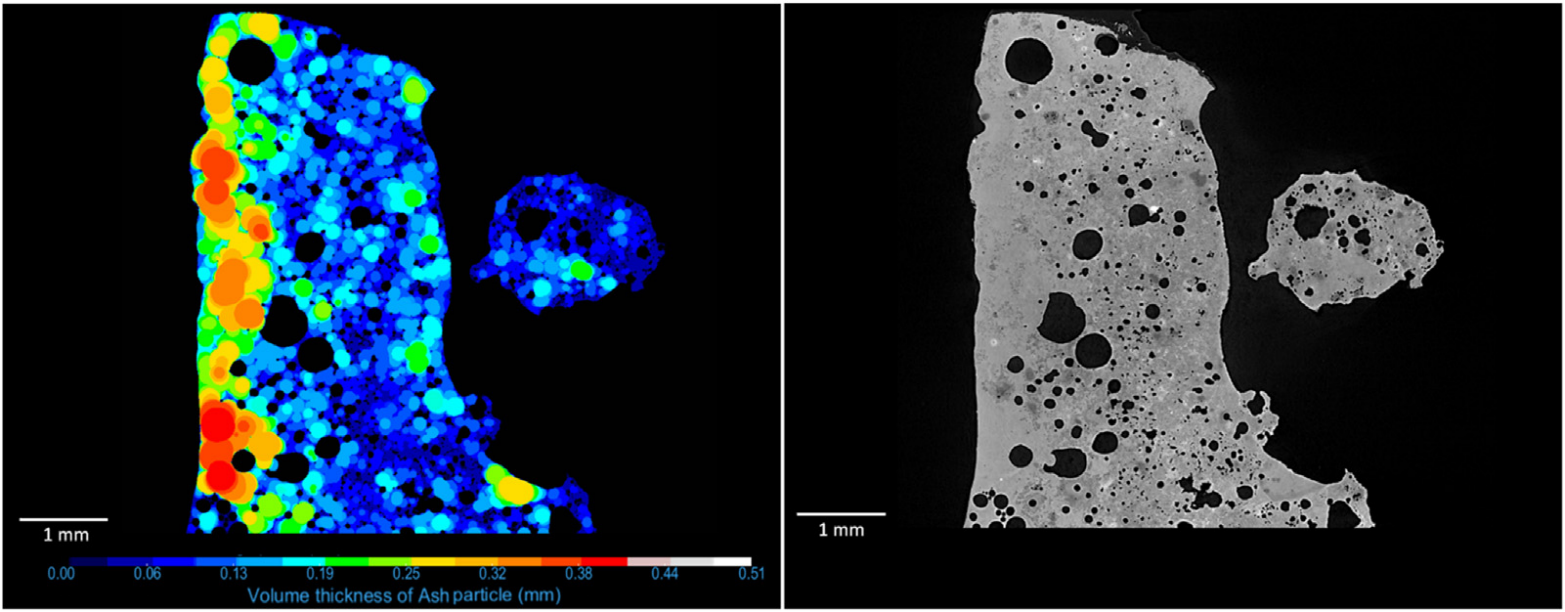
An ortho slice showing volume thickness map of the ash particle (left), coloured after intensity level that indicates the local thickness. To the right the corresponding ortho slice with the grey scale apply to X-ray attenuation intensities.

The “Ash particle” was used as ROI for wall thickness quantification, from which a volume thickness map was created. In the volume thickness map, each voxel has an intensity that indicates the local wall thickness for that voxel. The volume thickness map can be visualised and coloured after intensity (see Fig. 5). A new connectivity multi-ROI analysis was created from “Ash particle”, and by choosing the dataset of the volume thickness map in the Statistical properties box, the min, max and mean values of the intensity corresponding to the wall thickness can be calculated and exported.

### Specific surface area

**Fig. 6:**
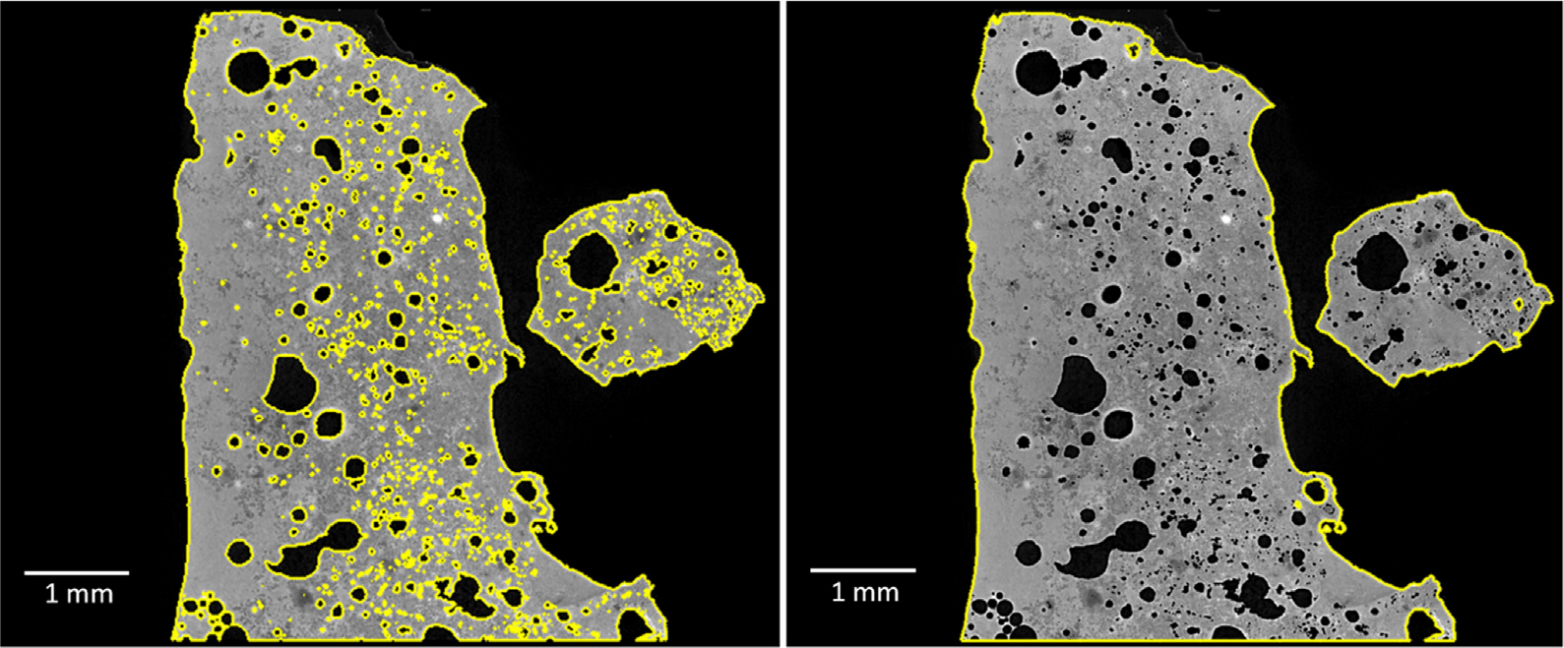
An ortho slice with a mesh in yellow to the left, visualising how the surface area is calculated, including the surface area of the discrete pores, and a mesh without the discrete pores to the right.

Two variants of surface area calculations were performed. With the first variant, an interpolated surface area of the labelled object (binary object) was calculated, in this case, the ROI “Ash particle”. A new connectivity multi-ROI analysis was created, and the surface area was calculated according to the method described by Lindblad [17]. This surface area refers to the outside of the object. However, this still includes, for example, all the pores in this project, visualised with a mesh in Fig. 6 (left).

A new ROI was created to avoid including the surface area of the discrete pores inside the particle, merging the ROI “Ash particle” and Discrete pores”. The surface area was then calculated by a new connectivity multi-ROI analysis; in practice, for this project, the open pores are included, but not the discrete pores (Fig. 6, right). The difference in this case was a 3.7 times higher surface area for the first variant. What variant of surface area calculation that is appropriate to use may depend on samples and area of use.

Specific surface area is common to use for porous materials. It's defined as the total surface area of an object per unit of mass or volume. The specific surface area was calculated by dividing the surface area by the volume of the ROI.

## Conclusion

A deep learning segmentation method was applied to X-ray microtomography data using the U-net architecture. The model was trained through a combination of manual input and iterative predictions/corrections to improve its accuracy. After deep learning training, the model could segment solid material (ash) and air (pores and background) of the ash particles. The deep learning approach solved the problem of features with similar intensities but different patterns, as well as managing intensity variations in the background, which are not so easy to get around with manual segmentation.

An approach for quantitative analysis of morphology, porosity, and internal microstructure of the samples was established. Porosity and open pore volume, pore size distribution and sphericity, particle wall thickness and specific surface area were generated.

## Method validation

**Fig. 7:**
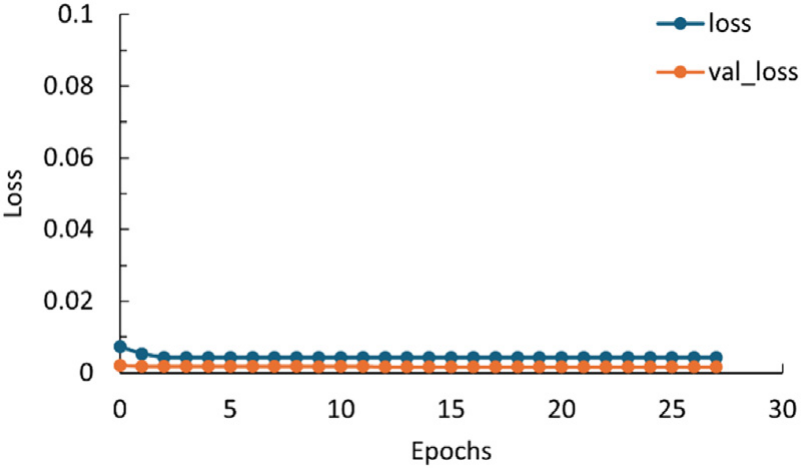
Loss and validation loss during training as a function of epochs.

To validate the deep learning model, the loss function ORS categorical cross-entropy was used. The ORS dice coefficient and the validation ORS dice coefficient was 0.997 respective 0.998 after 27 epochs, 1 being the maximum. The training was stopped after 27 epochs, due to no improvement in the validation loss in 15 consecutives epochs. In Fig. 7 the results of the training and validation losses can be seen, the training and validation loss are 0.0042 and 0.0017 after 27 epochs, respectively. A lower value is favourable, indicating a good accuracy of the model for prediction.

For further validation the method was applied to other ash samples. The method's segmentation part is validated. Comparing the segmented data to the training data gave scores between 0.9987 and 0.9994 for all tested samples, which shows the share of pixels predicted correctly.

The method works well for single ash particles. When tested on replicates of the same material, scanned with the same settings, the previously trained deep learning model could be applied without additional training with an acceptable result. However, results improved when a model trained explicitly for the new sample was used. It's important to note that training a new model becomes necessary when the material or settings are altered.

Due to the highly irregular shapes of the ash particles, assessing what constitutes open pores and the boundary to the background is a matter of judgment, and it is difficult to quantitatively validate its accuracy. However, the morphological operation “Close” and the operation “Fill inner areas” give the same results with repeated tests. To better validate what is open pores versus background further studies is needed, especially for other types of samples.

The results obtained from the quantitative analysis were compared to results obtained with the reference method presented in Strandberg et al. [8], see Table 1. The segmentation is better with the new method than the reference method, making direct comparison difficult. For example, the part of the sample containing glue at the edge could not be segmented within a reasonable time and had to be excluded with the reference method. It was about a third of the sample volume for sample 1 but none for sample 2.

**Table 1:** Results obtained from quantitative analysis with this new method and, as a comparison, results obtained with the reference method described in Strandberg et al. [8].

	Sample Porosity [vol%]		Open pore volume [vol%]		Mean eq. diameter of discrete pores [µm]		Average wall thickness [µm]		Specific surface area [mm^2^/mm^3^]	
	New	Reference	New	Reference	New	Reference	New	Reference	New	Reference
Sample 1	13.1	12.5	15.1	20.0	16.2	16.1	185.6	87.7	10.0	10.5
Sample 2	16.8	14.9	38.9	50.6	15.6	15.4	150.8	70.6	14.1	14.6

The main difference between AI-assisted deep learning at the reference method was open pore volume and the thickness of the particle walls. The open pore volume, which is significantly higher for the reference method, might depend on significant internal variations in the sample and the entire sample was not analysed for the reference method for sample 1, (parts excluded due to the glue). For sample 2, we have a substantial difference in the number of discrete pores, 154 000, compared to 116 000 for the reference method. This is due to the difference in the segmentation; there is a difference between what is segmented as ash and air inside the particle. With the new method, more pores could be segmented correctly. The difference in wall thickness is significant; partly, it may also be due to the sample volume, but it is also probably due to differences in the method. In this new method, the volume thickness map is used. In the other method, using the software Avizo 9.3, a distance map algorithm (Chamfer Distance Map) was used. The average thickness of the material between pores and particle surfaces was then calculated. By evaluating the volume thickness map (see Fig. 5) and compare with manual measurements, the assessment is that this new method gives the most accurate results.

## Limitations

While this method is effective for single ash particles, its performance is less satisfactory when multiple particles are analysed simultaneously. This can be the case if multiple particles have been scanned simultaneously, e.g. in a tube. The deep learning segmentation works well, but the method struggles when differentiating between open pores and background. The problem is the operation “Close”. The close operation can then be applied between two adjacent particles instead of just for open pores for separate particles. The operation “Fill inner areas” and subsequent quantification becomes difficult to implement satisfactorily.

## Ethics statements

The authors comply with the ethical guidelines of MethodsX.

## CRediT authorship contribution statement

**Anna Strandberg:** Conceptualization, Methodology, Software, Formal analysis, Investigation, Resources, Writing – original draft, Writing – review & editing, Visualization, Project administration, Funding acquisition. **Hubert Chevreau:** Conceptualization, Methodology, Writing – review & editing. **Nils Skoglund:** Conceptualization, Methodology, Resources, Writing – review & editing, Funding acquisition.

## Declaration of Competing Interest

The authors declare that they have no known competing financial interests or personal relationships that could have appeared to influence the work reported in this paper.

## Data Availability

Data will be made available on request.
